# A two-sample Mendelian randomization study of the causal relationship between respiratory diseases, gastric cancer risk, and *Helicobacter pylori* infection

**DOI:** 10.1007/s10120-026-01729-8

**Published:** 2026-03-06

**Authors:** Youjin Kim, Seunghyun Lee, Jongin Lee, Min Young Park, Jeehee Min, Xiaoxue Ma, Maiko Hajime Sumikawa, Jin-Ha Yoon, Wanhyung Lee

**Affiliations:** 1 Asan Medical Center, Seoul, Republic of Korea; 2 Department of Convergence Medicine, School of Medicine, Pusan National University, Yangsan, Republic of Korea; 3 Department of Occupational and Environmental Medicine, Seoul St. Mary’s Hospital, College of Medicine, The Catholic University of Korea, Seoul, Republic of Korea; 4 Department of Occupational and Environmental Medicine, College of Medicine, Hanyang University, Seoul, Republic of Korea; 5 Department of Pediatrics, The First Hospital of China Medical University, Shenyang, China; 6 The First Department of Internal Medicine, School of Medicine, University of Occupational and Environmental Health, Kitakyushu, Japan; 7 Department of Preventive Medicine, Institute of Occupational Medicine, Yonsei University College of Medicine, Seoul, Republic of Korea; 8 Department of Preventive Medicine, College of Medicine, Chung-Ang University, Heukseok-ro 84, Dongjak-gu, Seoul, 06974 Republic of Korea

**Keywords:** Gastric cancer, Respiratory diseases, Mendelian randomization, *Helicobacter pylori*, Gut-lung axis, Dust

## Abstract

**Background:**

Gastric cancer is a major global burden, yet evidence linking respiratory diseases to gastric cancer is limited. This study examined whether genetic susceptibility to respiratory diseases including asthma, chronic obstructive pulmonary disease (COPD), idiopathic pulmonary fibrosis (IPF), and lung diseases due to external agents (LDEA) was associated with gastric cancer and Helicobacter pylori infection, given shared immune and inflammatory pathways.

**Methods:**

We conducted a two-sample Mendelian randomization analysis using genetic variants as instrumental variables to assess causal associations between respiratory diseases and risk of gastric cancer and *H. pylori* infection. GWAS summary statistics for respiratory diseases were obtained from FinnGen, and for gastric cancer and *H. pylori* infection from the UK Biobank. Analyses were adjusted for confounders such as smoking and alcohol consumption. Sensitivity analyses evaluated robustness and assessed potential pleiotropy and heterogeneity among genetic variants.

**Results:**

No significant direct causal associations were found between respiratory diseases and gastric cancer risk. However, asthma and LDEA were significantly associated with increased risk of *H. pylori* infection. No associations were observed for COPD or IPF with either outcome. Sensitivity analyses indicated minimal pleiotropic or heterogeneity effects.

**Conclusion:**

This study investigated genetic susceptibility to respiratory diseases and their potential links to gastric cancer and *H. pylori* infection. While direct genetic evidence linking respiratory disease susceptibility to gastric cancer was limited, significant associations with *H. pylori* infection suggest possible indirect pathways involving respiratory diseases.

**Supplementary Information:**

The online version contains supplementary material available at 10.1007/s10120-026-01729-8.

## Introduction

Gastric cancer is a major global health concern, with particularly high prevalence in East Asian populations [1, 2]. Increasing attention has been given to the potential association between respiratory diseases and gastric cancer, although existing evidence remains limited and inconclusive [3–5]. Methodological challenges such as residual confounding and reverse causation have constrained traditional observational studies.

Mendelian randomization (MR), a robust causal inference method that uses genetic variants as instrumental variables (IVs), addresses these methodological limitations. MR reduces bias from confounding and reverse causation inherent in observational epidemiology [6, 7].

In this study, we focused on genetic susceptibility to several respiratory diseases related with gastric cancer that reported previously as exposures. Asthma, chronic obstructive pulmonary disease (COPD), idiopathic pulmonary fibrosis (IPF), and lung diseases due to external agents (LDEA) represent distinct inflammatory and injury responses in the lung [8–13]. These conditions may influence gastrointestinal health through the gut–lung axis, via mechanisms such as chronic systemic inflammation, immune modulation, and impaired mucociliary clearance, potentially increasing gastrointestinal exposure to inhaled particulates [14, 15].

Therefore, we conducted a MR analysis to evaluate the potential causal relationships between genetic liability to these respiratory diseases and gastric cancer outcomes, including gastric cancer risk and H. pylori infection status. Our aim was to determine whether shared genetic and immunological mechanisms underlying respiratory disease susceptibility also contribute to gastrointestinal disease vulnerability.

## Methods

### Study design

**Fig. 1:**
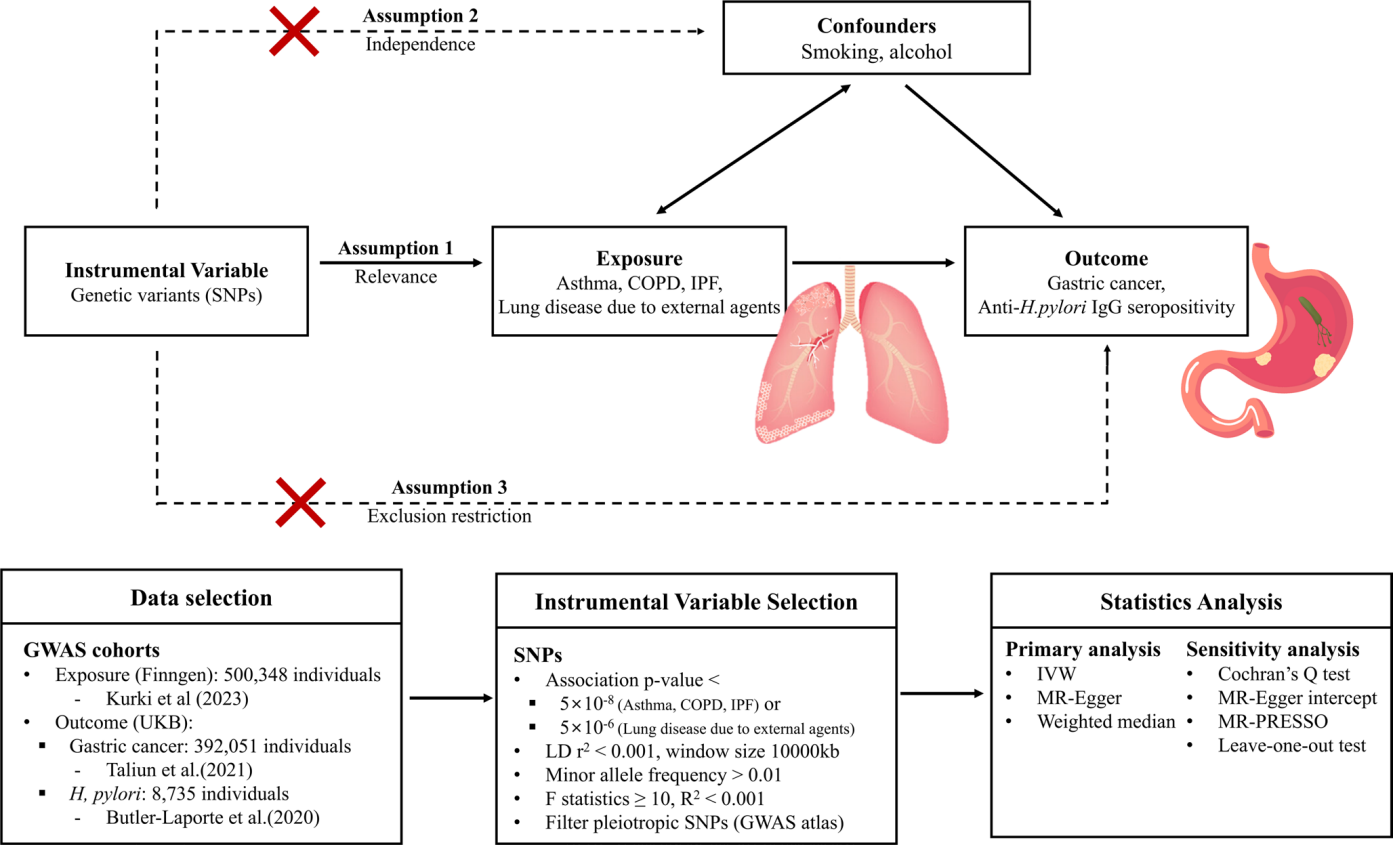
Overview of the current Mendelian randomization study

We conducted a two-sample MR analysis using publicly available summary-level statistics to investigate associations between respiratory diseases and gastric cancer, accounting for smoking as a confounder (see Fig. 1). MR relies on three core assumptions: (1) the exposure is associated with the genetic variants (relevance assumption); (2) the genetic variants and outcome have no shared unmeasured causes (independence assumption); and (3) the variants influence the outcome only through the exposure (exclusion restriction) [16]. We used the R package “TwoSampleMR” for the main analyses [17, 18] and “MRPRESSO” for sensitivity analyses [19].

### Data source

GWAS summary statistics were derived from individuals of European ancestry. For respiratory diseases, data were obtained from FinnGen (https://r12.finngen.fi/*)* [20], a public–private partnership integrating Finnish biobank genotype data with national health registries. Disease definitions followed ICD-10 codes: J45 and J46 for asthma (endpoint code: J10_ASTHMA_EXMORE), J43 and J44 for COPD (endpoint code: J10_COPD), J84.1 for IPF (endpoint code: IPF), and J60–J70 for LDEA (endpoint code: J10_EXTERLUNG), which includes lung injury from dusts, chemicals, and radiation (e.g., pneumoconiosis, hypersensitivity pneumonitis). Outcome summary statistics were obtained from the UK Biobank (UKB), a large prospective cohort of approximately 500,000 UK participants. Gastric cancer data (phecode 151) were accessed from Taliun et al. (https://pheweb.org/UKB-TOPMed/*)* [21], and anti-*H. pylori* IgG seropositivity data from Butler-Larpote et al. (GWAS ID: GCST90006910) [22]. In the GWAS of *H. pylori* IgG seropositivity, cases and controls were defined according to seropositivity to two or more *H. pylori* antigens. Seropositivity for each antigen was determined using antigen-specific median fluorescence intensity (MFI) thresholds recommended by the UK Biobank. Importantly, antibody titers were not analyzed as continuous quantitative traits; instead, we used a binary classification (seropositive vs. seronegative), which more directly reflects infection status. To account for potential confounders such as smoking and alcohol use, relevant associations were identified through the GWAS Catalog (https://www.ebi.ac.uk/gwas/*).* All GWAS summary statistics and variant positions were based on the GRCh38 (hg38) human genome build.

### Genetic instrument selection

We selected genetic instruments for asthma, COPD, and IPF using the conventional genome-wide significance threshold (*p* < 5 × 10^−8^), excluding SNPs associated with potential confounders. For LDEA, however, no SNPs surpassed this threshold due to the limited statistical power of the available GWAS. To ensure adequate instrument strength for this exposure, we adopted a more liberal threshold of *p* < 5 × 10^−6^, which has been applied in prior Mendelian randomization studies under similar circumstances [23, 24]. SNPs with r^2^ < 0.001 in linkage disequilibrium (LD) within a 10,000 kb window were excluded to ensure independence. LD reference panels were constructed from the 1000 Genomes Project for all populations [25, 26]. Non-biallelic, non-SNP, minor allele frequency < 0.01, and sex chromosome variants were excluded. We also removed SNPs with F-statistics < 10 and R^2^ < 0.001, as these are considered weak instruments. After harmonization, palindromic SNPs with intermediate allele frequencies were excluded. The remaining SNPs were included in the MR analysis.

### MR analysis

We applied the fixed-effect inverse variance weighting (IVW), MR-Egger, and weighted median methods. Specifically, fixed-effect and multiplicative random-effects IVW estimates were obtained using the mr_ivw_fe and mr_ivw_mre functions in the TwoSampleMR package, respectively. The choice of a fixed-effect IVW model was based on the absence of statistically significant heterogeneity across genetic instruments, as assessed by Cochran’s Q statistic (all *p* > 0.05). In this context, the fixed-effect IVW approach provides the most efficient and precise estimate. To ensure robustness, we additionally performed multiplicative random-effects IVW analyses, which yielded results that were consistent in both direction and significance with the fixed-effect estimates. IVW aggregates the effects of multiple IVs [24] but is sensitive to pleiotropic bias. To address this, we also used MR-Egger, which adjusts for directional pleiotropy [27], and the weighted median method, which yields consistent results even if up to 50% of IVs are invalid [28]. All results were reported as odds ratios (OR).

Sensitivity analyses were conducted for significant results. Cochran’s Q statistics assessed heterogeneity [28]. MR-Egger regression [27] and MR-PRESSO [19] evaluated pleiotropy and identified potential outlier SNPs.

## Results

The final number of SNPs used for respiratory diseases ranged from 7 to 71. Due to differences in SNP coverage across outcome GWAS datasets, the number of IVs included in each analysis varied slightly, although the same exposure data were used (Table 1). In MR analyses evaluating the causal relationship between respiratory diseases (FinnGen) and gastric cancer (UK Biobank), none of the conditions (asthma, COPD, IPF, LDEA) demonstrated statistically significant associations with gastric cancer risk by any MR method (IVW, MR-Egger, weighted median). Specifically, IVW yielded odds ratios (OR) of 1.00 (95% CI 0.74–1.36) for asthma, 1.19 (95% CI 0.83–1.72) for COPD, 1.06 (95% CI 0.79–1.42) for IPF, and 1.06 (95% CI 0.79–1.42) for LDEA. However, when evaluating associations with anti-*H. pylori* IgG seropositivity, significant findings emerged. Asthma was significantly associated with increased *H. pylori* seropositivity in the IVW analysis (OR: 1.21, 95% CI 1.01–1.44) and showed marginal significance in the weighted median method (OR: 1.28, 95% CI 1.00–1.64). LDEA also showed a significant association with *H. pylori* seropositivity in the IVW method (OR: 1.36, 95% CI 1.03–1.79), though this result was not supported by MR-Egger or weighted median analyses, warranting caution in interpretation. No significant associations were found for COPD or IPF with *H. pylori* seropositivity in any analytical approach. The detailed results of the IVW fixed-effect and multiplicative random-effects analyses, together with heterogeneity statistics, are provided in Supplementary Table 1.

**Table 1:** Mendelian randomization estimates for associations between lung diseases and the risk of gastric cancer or H. pylori infection

Exposure	Outcome	No. of SNPs	Odds ratio (95% confidence interval)		
			IVW-MR	MR-Egger	Weighted median
Asthma	Gastric cancer	71	1.00 (0.74–1.36)	0.97 (0.42–2.23)	0.89 (0.57–1.38)
COPD		21	1.19 (0.83–1.72)	0.76 (0.35–1.65)	0.94 (0.56–1.58)
IPF		14	1.06 (0.79–1.42)	1.21 (0.82–1.79)	1.17 (0.79–1.72)
LDEA		10	1.06 (0.79–1.42)	1.21 (0.82–1.79)	1.17 (0.79–1.72)
Asthma	Anti-*H. pylori* IgG seropositivity	68	**1.21 (1.01–1.44)**	1.34 (0.81–2.23)	**1.28 (1.00–1.64)**
COPD		22	0.97 (0.80–1.19)	0.73 (0.47–1.12)	0.89 (0.67–1.17)
IPF		12	1.07 (0.90–1.26)	1.46 (0.96–2.22)	1.06 (0.85–1.33)
LDEA		7	**1.36 (1.03–1.79)**	0.87 (0.45–1.67)	1.27 (0.89–1.82)

Bold values indicate statistically significant associations

IVW, inverse-variance weighted; COPD, chronic obstructive pulmonary disease; IPF, idiopathic pulmonary fibrosis; LDEA, lung diseases due to external agents

**Fig. 2:**
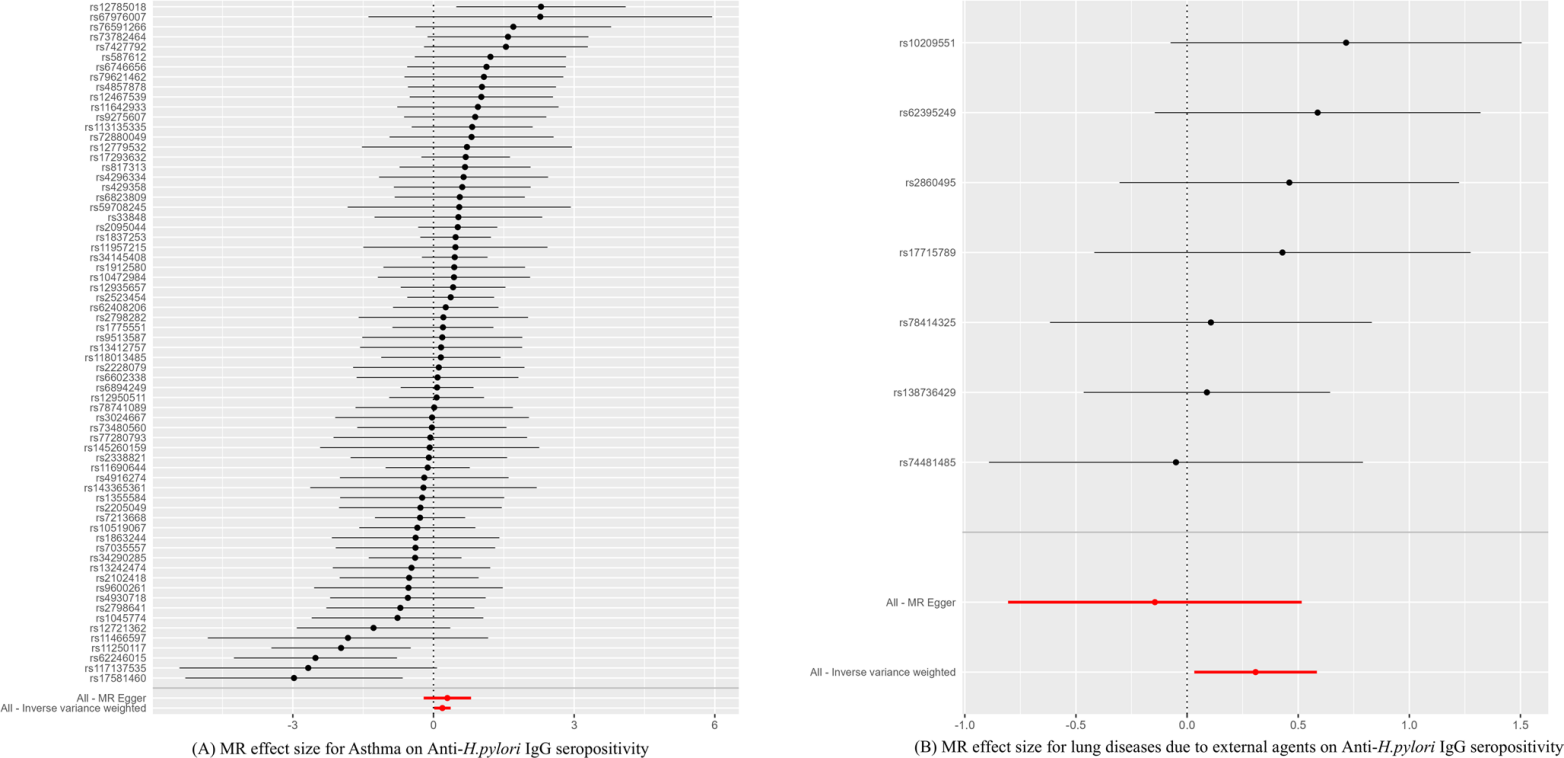
Forest plots summarizing the Mendelian randomization analysis results for **A** asthma and **B** lung diseases due to external agents, in relation to anti-*H. pylori* IgG seropositivity. Black dots indicate odds ratios (ORs); horizontal lines denote 95% confidence intervals (CIs). The vertical black dashed line represents OR = 1; values greater than 1 indicate a positive association between exposure and outcome, and values less than 1 indicate a negative association

**Fig. 3:**
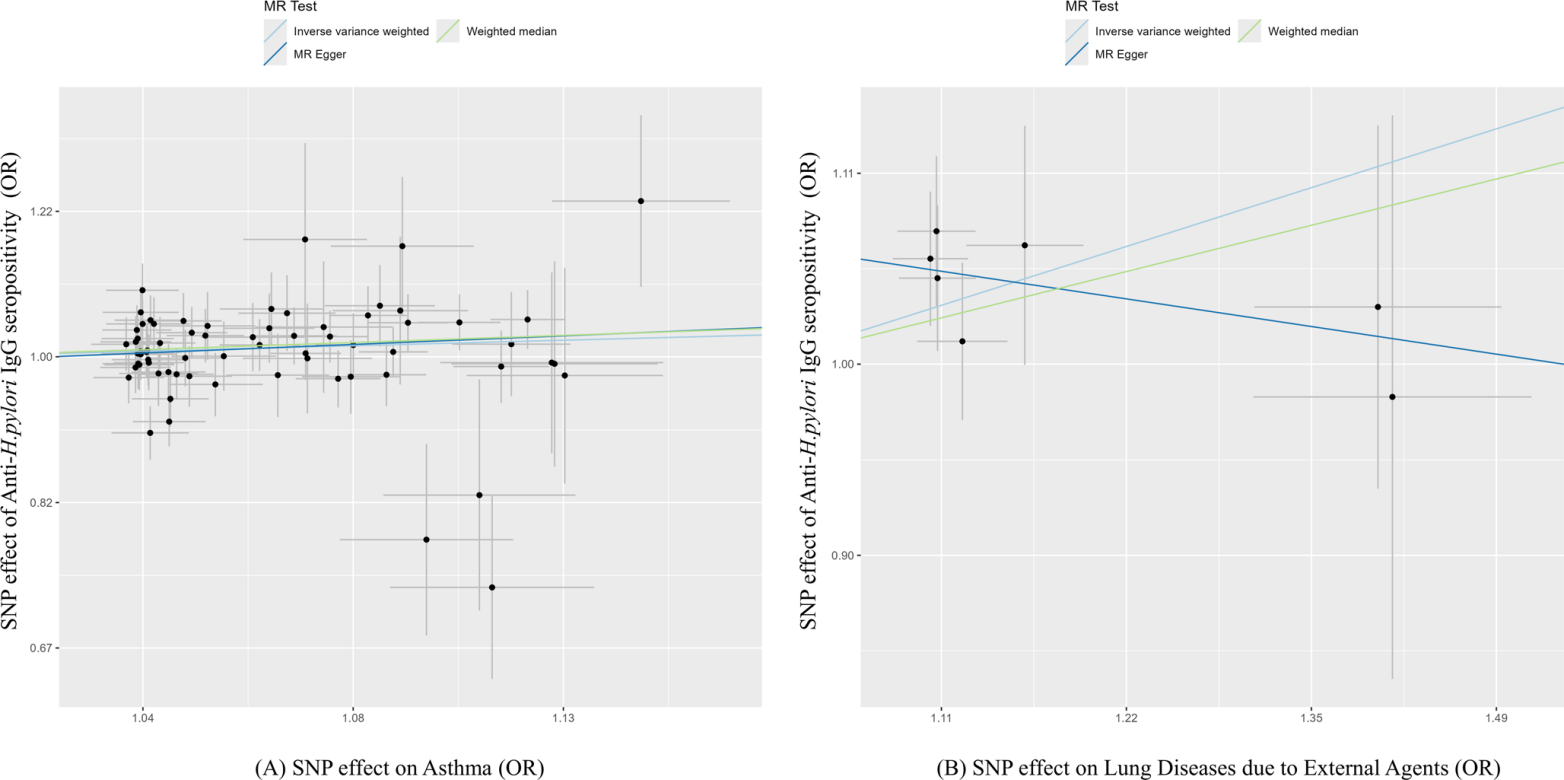
Scatter plots depicting the Mendelian randomization effects (odds ratio and 95% confidence intervals) of **A** asthma and **B** lung diseases due to external agents on *H. pylori* IgG seropositivity. The X-axis shows SNP effects on respiratory diseases, and the Y-axis shows SNP effects on *H. pylori* IgG seropositivity. The three slanted lines correspond to the three Mendelian randomization analysis methods. Each black dot represents a single nucleotide polymorphism

Extensive sensitivity analyses—including Cochran’s Q test, MR-Egger intercept test, and MR-PRESSO analysis—revealed no substantial heterogeneity or directional pleiotropy, supporting the robustness and reliability of the MR results (Table 2). SNP-level effect estimates, reflecting individual genetic instrument contributions, are visually presented as forest plots (Fig. 2), leave-one-out analyses (Supplementary Fig. 1), and scatter plots (Fig. 3). Detailed information on the instrumental SNPs used in these analyses is provided in Supplementary Table 2.

**Table 2:** Sensitivity analysis of the association between lung diseases and risk of gastric cancer or *H. pylori* infection

Exposure	Outcome	Cochrane’s Q		*P*_MR−PRESSO_	*P*_intercept_
		Q value	*P*_Q_		
Asthma	Gastric cancer	75.520	0.305	0.258	0.940
COPD		15.858	0.725	0.540	0.210
IPF		13.283	0.426	0.482	0.646
LDEA		11.125	0.267	0.275	0.351
Asthma	Anti-*H. pylori* IgG seropositivity	71.646	0.326	0.331	0.661
COPD		16.771	0.725	0.752	0.160
IPF		7.560	0.752	0.755	0.140
LDEA		3.403	0.757	0.762	0.199

IVW, inverse-variance weighted; COPD, chronic obstructive pulmonary disease; IPF, idiopathic pulmonary fibrosis; LDEA, lung diseases due to external agents

For transparency of instrument strength, we listed all SNP instruments with their association statistics for both the exposure and the outcome. Specifically, for each variant we report the effect allele, reference allele, chromosome position (GRCh38), effect size and standard error for the association with the exposure, as well as the corresponding association statistics with the outcome (*H. pylori* seropositivity or gastric cancer). These data are provided in Supplementary Table 3.

Together, these findings demonstrate that while genetically inferred causal relationships between respiratory diseases and gastric cancer were not observed, certain respiratory conditions—notably asthma and LDEA—showed significant genetic associations with the risk of *H. pylori* infection, suggesting potential indirect biological pathways via the gut–lung axis.

## Discussion

In this study, we conducted an MR analysis to examine whether genetic susceptibility to respiratory diseases influences the risk of gastric cancer and *H*. *pylori* infection. Our results did not indicate a genetically inferred causal relationship between respiratory disease susceptibility and gastric cancer risk. However, we found significant genetic associations between susceptibility to specific respiratory diseases notably asthma and LDEA and an increased risk of *H. pylori* infection. These findings suggest that genetic predispositions underlying respiratory conditions may overlap with genetic susceptibility to *H. pylori* infection, potentially mediated by shared biological mechanisms such as systemic inflammation or immune modulation. Thus, our results emphasize the importance of considering genetic susceptibility pathways when interpreting relationships between respiratory diseases and gastrointestinal conditions.

Asthma and LDEA may influence gastrointestinal health through several plausible biological mechanisms and potentially contribute to *H. pylori* colonization and persistence. These respiratory diseases aggravate pulmonary mucosal immune responses, leading to chronic inflammation characterized by increased systemic levels of pro-inflammatory cytokines and chemokines [29, 30]. This systemic inflammation may compromise gastrointestinal mucosal barrier integrity, alter local immune defenses, and increase susceptibility to microbial infections [13, 31]. Additionally, asthma and LDEA could disrupt mucociliary clearance, resulting in increased microaspiration of particulates into the gastrointestinal tract and directly affecting the gastric mucosa [32, 33]. Long-term dust exposure may also induce dysbiosis by altering gut microbiota composition, further impacting mucosal immunity and promoting conditions favorable to *H. pylori* infection [34].

Our study found that asthma is significantly associated with an increased risk of *H. pylori* infection. This association may be attributable to asthma-related systemic inflammation, changes in mucosal immunity, and disruptions in gut microbiota balance, all of which could predispose individuals to gastrointestinal infections [35]. Previous studies have shown that chronic inflammatory states, such as those in asthma, can impair gastrointestinal mucosal integrity, facilitating *H. pylori* colonization and persistence [36]. Furthermore, immune dysregulation commonly observed in individuals with asthma—including an overactive T-helper 2 cytokine profile with elevated IL-4, IL-5, and IL-13, which downregulate tight-junction proteins and increase intestinal epithelial permeability—may facilitate bacterial colonization and impair mucosal clearance [37, 38]. However, asthma is a complex and multifactorial disease, influenced by occupational and environmental dust exposures as well as genetic predisposition, allergens, viral infections, and lifestyle factors. Therefore, although our findings support a potential role for asthma in modulating gastric mucosal conditions through immune or microbiota-related pathways, they do not conclusively support the primary hypothesis that occupational and environmental dust exposure directly or predominantly mediates gastric cancer risk via respiratory conditions. Additional research with well-characterized exposure histories and more targeted analyses is needed to clarify the specific contributions of environmental dust exposure to the observed associations.

LDEA more directly reflects chronic occupational or environmental dust exposure compared to asthma or other lung diseases. The association between LDEA and increased *H. pylori* infection observed in our study provides stronger evidence for the hypothesis that inhaled occupational and environmental dust may indirectly affect gastric conditions through respiratory pathways. LDEA involves respiratory impairments resulting from prolonged inhalation of external dust and particulate matter, often marked by chronic inflammation, impaired mucociliary clearance, and lung tissue damage [39]. These pathological features may facilitate the direct transport or prolonged gastrointestinal exposure to inhaled particulates, potentially disrupting mucosal integrity and predisposing individuals to microbial colonization, such as *H. pylori* [40, 41]. Thus, the findings regarding LDEA more directly support the potential role of occupational and environmental dust exposures in modulating gastrointestinal disease risk through the gut–lung axis. Nonetheless, because statistical significance was observed only in the IVW-MR analysis and not in the MR-Egger or Weighted Median approaches, this finding should be interpreted cautiously. The inconsistency among MR methods suggests possible residual pleiotropy or heterogeneity in the genetic instruments, underscoring the need for cautious interpretation and further validation in future studies using refined methodologies. Moreover, the diagnostic criteria for LDEA are relatively nonspecific, potentially encompassing a range of etiologies and clinical presentations, which warrants additional caution in interpreting its role as a direct proxy for occupational and environmental dust exposure.

COPD and IPF were not significantly associated with either gastric cancer or *H. pylori* infection in our analysis. This absence of association may reflect distinct pathophysiological features that differentiate these diseases from asthma and LDEA. COPD is characterized primarily by chronic inflammation with neutrophilic infiltration, oxidative stress, and airway remodeling, rather than the type 2 immune responses typical of asthma [42]. These inflammatory pathways in COPD may not substantially affect gastrointestinal mucosal immunity or microbiota composition, limiting their influence on gastric mucosal susceptibility to infections such as *H. pylori*. Similarly, IPF is predominantly defined by fibroproliferative processes and extracellular matrix deposition rather than persistent inflammatory responses [43], which may explain its limited impact on gastrointestinal health via the gut–lung axis. Therefore, these differences in immunological profiles and disease mechanisms likely underlie the absence of associations in our MR analysis, highlighting the relevance of disease-specific mechanisms in respiratory–gastrointestinal interactions.

Previous studies have shown that *H. pylori* antibody titers vary depending on infection persistence or treatment outcomes. For example, individuals who fail eradication treatment tend to retain higher antibody levels over time, whereas those with successful eradication show greater decreases in titers [44, 45]. These findings support the notion that antibody titers can reflect not only infection status but also its persistence and intensity. Although our analysis employed a binary seropositivity definition based on antigen-specific thresholds rather than continuous antibody titers, this approach is consistent with prior evidence that antibody positivity reflects infection-related risk and not merely transient immune activation.

Given the potential indirect pathways linking respiratory health and *H. pylori* infection through systemic inflammation and mucosal immune disruption, our findings suggest that attention to respiratory health may have broader implications for gastrointestinal outcomes. While the evidence remains preliminary, exploring whether effective management of respiratory diseases could also help reduce susceptibility to *H. pylori* colonization is an important direction for future research. Further empirical studies are needed to clarify these mechanisms and to evaluate their potential relevance for preventive or clinical strategies.

This study had several limitations. First, MR analysis depends on the validity of IVs. Although we selected genetic variants associated with respiratory diseases, these conditions particularly asthma have multifactorial etiologies and may not exclusively reflect shared pathways relevant to gastric cancer. Therefore, caution is warranted in interpreting these results. Second, our genetic instruments were largely derived from GWAS conducted in European populations, which may limit the generalizability of our findings to East Asian populations who have different genetic backgrounds and higher gastric cancer prevalence. Third, although our MR analyses suggested potential associations, LD Score regression based genetic correlation analyses did not demonstrate significant shared heritability between respiratory diseases and gastric cancer or *H. pylori* infection. These findings suggest that caution is warranted in interpreting the MR associations as evidence of a shared genetic architecture. The observed MR results may reflect mechanisms beyond additive genome-wide correlations, such as disease-specific immune pathways or environmental interactions. Finally, LDEA’s diagnostic categorization is inherently nonspecific, encompassing various diseases arising from diverse particulate exposures and introducing potential heterogeneity and misclassification bias. Future studies should incorporate precisely characterized exposure data, diverse populations, and refined diagnostic criteria to confirm these initial findings.

In summary, our study investigated whether genetic susceptibility to respiratory diseases is associated with gastric cancer risk and *H. pylori* infection. While direct evidence linking respiratory conditions to gastric cancer remains scarce, our findings suggest potential indirect pathways mediated by immune and inflammatory mechanisms. These results highlight that respiratory health may play an important role in shaping genetic susceptibility to gastrointestinal disease. Future research should further explore these associations to clarify underlying biological mechanisms and to identify practical strategies for prevention and treatment.

## Supplementary Information

Below is the link to the electronic supplementary material.

Supplementary Material 1

Supplementary Material 2

Supplementary Material 3

Supplementary Material 4

## Data Availability

Not available.
