# Supplementary material for: A two-sample Mendelian randomization study of the causal relationship between respiratory diseases, gastric cancer risk, and Helicobacter pylori infection
Source: Gastric Cancer. 2026 Mar 6;29(3):519–26. doi: 10.1007/s10120-026-01729-8 (PMC13124861; doi:10.1007/s10120-026-01729-8)
Supplement: Supplementary file 3 — Supplementary Material 3 [file 10120_2026_1729_MOESM3_ESM.docx]

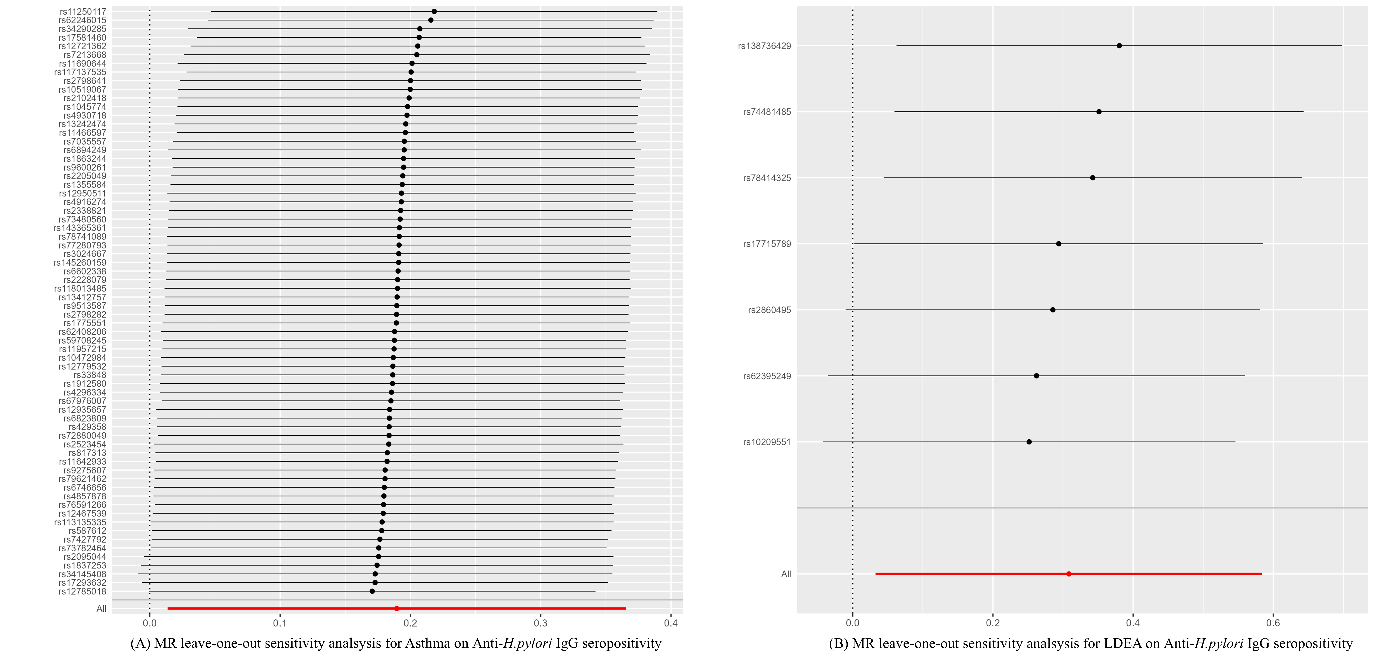


Supplementary Figure 1. Leave-one-out plots summarizing the Mendelian randomization analysis results for (A) asthma and (B) lung diseases due to external agents (LDEA) in relation to anti-*H. pylori* IgG seropositivity are shown. Black dots indicate odds ratios (OR), with horizontal lines denoting 95% confidence intervals (CI). The vertical black dashed line indicates OR = 1; values above 1 suggest a positive association between exposure and outcome, whereas values below 1 suggest a negative association.
