# Supplementary material for: A two-sample Mendelian randomization study of the causal relationship between respiratory diseases, gastric cancer risk, and Helicobacter pylori infection
Source: Gastric Cancer. 2026 Mar 6;29(3):519–26. doi: 10.1007/s10120-026-01729-8 (PMC13124861; doi:10.1007/s10120-026-01729-8)
Supplement: Supplementary file 4 — Supplementary Material 4 [file 10120_2026_1729_MOESM4_ESM.docx]

Supplementary Table 2. SNPs used as instrumental variables for asthma and lung diseases due to external agents

| RSID | Chromosome | Position | Reference allele | Alternative allele | Nearest gene | P-value |
| --- | --- | --- | --- | --- | --- | --- |
| **Asthma** |  |  |  |  |  |  |
| rs11466597 | 1 | 91719794 | T | C | TGFBR3 | 1.27E-08 |
| rs4916274 | 1 | 172794355 | T | A | RP1-15D23.2 | 7.96E-10 |
| rs2228079 | 1 | 203129147 | T | G | ADORA1 | 2.16E-08 |
| rs13412757 | 2 | 8317950 | G | A | LINC00299 | 6.63E-10 |
| rs77280793 | 2 | 47095617 | C | T | RP11-761B3.1, STPG4 | 2.77E-08 |
| rs12467539 | 2 | 100785893 | C | T | NPAS2 | 5.83E-11 |
| rs11690644 | 2 | 102297754 | A | G | IL1RL1 | 1.67E-32 |
| rs113135335 | 2 | 111130177 | T | G | BCL2L11, MIR4435-2HG | 4.49E-16 |
| rs6746656 | 2 | 173076286 | G | A | MAP3K20 | 7.64E-09 |
| rs34290285 | 2 | 241759225 | G | A | D2HGDH | 1.24E-21 |
| rs7427792 | 3 | 57756998 | G | T | SLMAP | 4.14E-08 |
| rs62246015 | 3 | 71432041 | C | T | FOXP1, RP11-79P21.2 | 1.82E-08 |
| rs4857878 | 3 | 128229306 | T | C | EEFSEC | 5.85E-11 |
| rs587612 | 3 | 193819770 | G | A | LINC02038 | 1.22E-09 |
| rs2798282 | 4 | 3028189 | A | G | GRK4 | 3.88E-09 |
| rs17581460 | 4 | 38454783 | T | C | LINC01258 | 4.28E-08 |
| rs6823809 | 4 | 105907638 | T | C | NPNT | 1.94E-12 |
| rs10472984 | 5 | 35843730 | C | G | IL7R | 1.28E-10 |
| rs11957215 | 5 | 40445579 | T | C | RP11-386E5.1 | 3.52E-08 |
| rs73782464 | 5 | 110407391 | T | C | TMEM232 | 1.64E-15 |
| rs1837253 | 5 | 111066174 | T | C | TMEM232 | 1.35E-37 |
| rs76591266 | 5 | 111219544 | A | T | CAMK4 | 4.79E-11 |
| rs59708245 | 5 | 130382674 | A | C | RNU7-53P | 1.17E-08 |
| rs6894249 | 5 | 132461855 | A | G | AC116366.7, IRF1, IRF1-AS1 | 9.18E-41 |
| rs2338821 | 5 | 142129868 | A | G | NDFIP1 | 3.11E-09 |
| rs2523454 | 6 | 31400088 | G | A | MICA | 1.69E-26 |
| rs34145408 | 6 | 32634347 | C | T | HLA-DQA1 | 6.33E-58 |
| rs9275607 | 6 | 32715868 | A | G | XXbac-BPG254F23.7 | 2.94E-13 |
| rs72880049 | 6 | 33525062 | G | A | GGNBP1 | 8.64E-09 |
| rs62408206 | 6 | 90161868 | G | A | BACH2 | 3.74E-16 |
| rs2798641 | 6 | 108946847 | C | T | ARMC2 | 2.81E-09 |
| rs79621462 | 6 | 151932980 | A | G | ESR1 | 1.17E-09 |
| rs13242474 | 7 | 20401442 | A | G | ITGB8 | 1.90E-10 |
| rs11250117 | 8 | 11115230 | C | A | XKR6 | 6.45E-11 |
| rs2102418 | 8 | 80383509 | G | T | RP11-941H19.3 | 5.39E-11 |
| rs2095044 | 9 | 6192796 | T | C | GTF3AP1 | 8.81E-33 |
| rs7035557 | 9 | 6611329 | G | A | GLDC | 2.08E-11 |
| rs1045774 | 9 | 124270328 | A | G | NEK6 | 2.49E-08 |
| rs78741089 | 9 | 129178466 | G | C | RP11-344B5.4, RP11-344B5.6 | 2.74E-08 |
| rs117137535 | 9 | 137605991 | G | A | ARRDC1, RP11-48C7.8 | 1.20E-08 |
| rs12785018 | 10 | 8473385 | C | T | RP11-543F8.3 | 7.41E-09 |
| rs6602338 | 10 | 8796002 | T | C | RP11-575N15.1 | 5.31E-09 |
| rs1912580 | 10 | 8995019 | G | C | RP11-428L9.2 | 5.86E-10 |
| rs1775551 | 10 | 9011080 | C | A | RP11-428L9.2 | 1.14E-28 |
| rs12779532 | 10 | 21650129 | C | T | MLLT10 | 4.91E-12 |
| rs1863244 | 11 | 10636192 | C | T | IRAG1 | 9.33E-09 |
| rs73480560 | 11 | 57633927 | C | T | AP000662.4 | 3.11E-11 |
| rs145260159 | 11 | 76711815 | T | G | GUCY2EP | 1.69E-09 |
| rs12721362 | 12 | 47831959 | T | C | HDAC7, LINC02354 | 6.13E-10 |
| rs4930718 | 12 | 123428886 | A | G | RILPL2 | 2.04E-09 |
| rs9600261 | 13 | 74325496 | G | A | RP11-512C24.4 | 9.45E-09 |
| rs9513587 | 13 | 99272114 | C | T | UBAC2 | 2.04E-09 |
| rs10519067 | 15 | 60776148 | G | A | RORA | 1.18E-17 |
| rs17293632 | 15 | 67150258 | C | T | SMAD3 | 3.92E-29 |
| rs1355584 | 15 | 70318066 | C | T | RP11-543G18.1 | 8.88E-09 |
| rs12935657 | 16 | 11125184 | G | A | CLEC16A | 1.86E-17 |
| rs3024667 | 16 | 27360263 | T | C | IL4R | 6.66E-09 |
| rs11642933 | 16 | 29935387 | G | A | TMEM219 | 3.08E-09 |
| rs7213668 | 17 | 39910256 | G | A | GSDMB | 1.78E-30 |
| rs12950511 | 17 | 49243576 | C | T | FLJ40194 | 5.52E-26 |
| rs4296334 | 18 | 23577175 | C | T | NPC1 | 1.35E-08 |
| rs118013485 | 19 | 33235671 | G | A | SLC7A10 | 9.99E-23 |
| rs33848 | 19 | 33533344 | G | A | PEPD | 9.49E-09 |
| rs429358 | 19 | 44908684 | T | C | APOE | 8.29E-14 |
| rs67976007 | 20 | 24379863 | G | A | RP4-564O4.1 | 1.70E-09 |
| rs817313 | 20 | 63951034 | T | G | UCKL1 | 2.83E-13 |
| rs143365361 | 21 | 35045948 | T | A | RUNX1 | 1.21E-10 |
| rs2205049 | 21 | 35323611 | T | C | RUNX1 | 2.99E-09 |
| **Lung diseases due to external agents** | | |  |  |  |  |
| rs10209551 | 2 | 69770863 | A | G | ANXA4 | 4.03E-06 |
| rs62395249 | 5 | 179554365 | C | T | RUFY1 | 3.89E-06 |
| rs138736429 | 6 | 130798763 | A | C | SMLR1 | 4.81E-07 |
| rs74481485 | 9 | 4527103 | T | C | SLC1A1 | 4.74E-06 |
| rs2860495 | 10 | 103515485 | T | C | NEURL1, NEURL1-AS1 | 2.52E-06 |
| rs17715789 | 16 | 5904314 | A | G | RBFOX1 | 4.61E-06 |
| rs78414325 | 18 | 11659404 | C | T | MIR7153 | 4.81E-06 |
